# Case Report: Food-based enteral formula in the nutritional management of children with hyperinsulinism: single center retrospective case series

**DOI:** 10.3389/fendo.2025.1574093

**Published:** 2025-09-25

**Authors:** Graeme O’Connor, Lauren Cheng, Annaruby Cunjamalay

**Affiliations:** Dietetic Department, Great Ormond Street Hospital for Children National Health Service (NHS) Foundation Trust, London, United Kingdom

**Keywords:** hyperinsulinism, food-based enteral formula, gastrointestinal tolerance, food-derived ingredients, glucose control, fiber

## Abstract

**Context:**

Hyperinsulinism is characterized by dysregulated insulin secretion and is typically associated with reduced fasting tolerance. Long-term hyperinsulinism management includes nutrition and medication to normalize plasma glucose levels. Management of enteral feeds and oral intake is integral to the clinical management strategy in children with hyperinsulinism. A new generation of commercial Food Based Enteral Formulas (FBF) consists of rehydrated food, whole‐food protein sources and a mixture of soluble and insoluble fiber.

**Objective:**

Review the tolerance of a food-based enteral formula for the nutritional management of children with hyperinsulinism.

**Methods:**

A single-center retrospective case series to explore the dietetic practice of prescribing a commercially available FBF (Compleat^®^ pediatric, Nestlé Health Science) in children with congenital hyperinsulinism (CHI) or postprandial hypoglycemia (PPH). Data were collected on demographics, gastrointestinal symptoms, anthropometrics, percentage glucose management indicators (GMI%) and hypo/hyperglycemic episodes.

**Results:**

Data were collected on eight children; six were diagnosed with CHI and two children had PPH. The mean age was 1.4 years (0.4SD). All children had a gastrostomy for enteral nutrition. Six of the eight children were prescribed proton pump inhibitors or stool softeners to manage gastroesophageal reflux and/or constipation symptoms. Within one month of children being prescribed FBF dietitians documented an 80% improvement in gastrointestinal symptoms. Dexcom glucose data was available for six children. Six months after FBF was prescribed, Dexcon Inc. data reported that five children had an improvement or stable GMI% and all six children had a reduction in hyperglycemic episodes (chi-square =5.8, p-value 0.02). Children prescribed FBF were less likely to require a glucose polymer (Chi-square = 4.9, p-value = 0.02).

**Conclusion:**

Our retrospective case series suggests that FBF is well tolerated in children with hyperinsulinism and may mitigate gut motility issues associated with hyperinsulinism, especially in relation to gastro-esophageal reflux and constipation symptoms. Furthermore, FBF may help reduce the dependency on glucose polymers and reduce the likelihood of a hyperglycemic episode. A larger sample size and longer follow-up study are necessary to substantiate the potential beneficial impact of FBF’s on glucose control in children with hyperinsulinism.

## Highlights

- FBF are a new generation of enteral formulas with limited real-world evidence
- FBF may improve gastroesophageal reflux and constipation in children with CHI
- FBF may reduce the dependency on glucose polymers

## 1 Introduction

Hyperinsulinism is characterized by unregulated and inappropriate insulin secretion from pancreatic β-cells. It results in hypoglycemia and is the most common cause of persistent hypoglycemia in neonates (1). For this case series, we will specifically focus on Congenital hyperinsulinism (CHI) and post-prandial hypoglycemia (PPH). CHI is a genetically and morphologically heterogeneous group of disorders associated with persistent dysregulated insulin secretion, associated with a significant risk of epilepsy, causing permanent brain damage. The molecular basis involves defects in key genes. The most common mutations affect the potassium adenosine triphosphate (K-ATP) channel genes ABCC8 and KCNJ11, which regulate insulin secretion. Rapid genetic analysis, imaging with 18F-DOPA-PET/CT scan, and development in surgical techniques have improved the management and outcome of the disease (2). Reactive hypoglycemia is a condition of PPH that occurs 2–5 hours after food intake. Elevated insulin levels usually account for hypoglycemia. Some patients rarely show increased insulin sensitivity (3).

The long-term management of hyperinsulinism involves medication to manage the inappropriate over-secretion of insulin through drugs like diazoxide, octreotide, and nifedipine (4). Treatment strategies are individualized based on the specific type and cause of hyperinsulinism, with the aim to normalize plasma glucose levels, provide an age-adjusted fasting tolerance and avoid neurological symptoms associated with hypoglycemia (5). Nutritional management is a crucial component in the treatment of hyperinsulinism, particularly in preventing and managing hypoglycemic episodes. The nutritional strategies may include frequent feeding with high-energy carbohydrate feeds and reducing the frequency and severity of hypoglycemic episodes (2). Children may require additional carbohydrates, which can be delivered as glucose polymers, high-energy formulas, and energy-dense foods. It is recommended that children requiring additional oral carbohydrates to prevent hypoglycemia should be referred to an experienced pediatric dietitian (6).

The current evidence supports the use of dietary fiber in enteral feeding formulas as a first-line therapy for children who need nutritional support (7). Soluble fiber products and fiber from natural foods are effective in improving glycemic control and insulin sensitivity. The magnitude of blood glucose control depends on the fiber type, dose, and intervention length (8). Furthermore, it is recognized that specific dietary components, such as certain dietary fibers, can increase the abundance of specific gut bacteria, which is beneficial for glucose regulation. Dietary fiber improves glucose metabolism and is associated with an increased abundance of the gut microbiome that protects against Bacteroides, which can induce glucose intolerance (9, 10).

Commercial food‐based enteral formulas are rapidly growing in popularity and may help with gastrointestinal symptoms such as constipation, reflux, and loose stools. A new generation of commercial Food Based Formulas (FBFs) is made with a variety of whole‐food protein sources, such as pea protein and chicken, and a mixture of soluble and insoluble fiber (1g per 100ml enteral formula). The development of commercial FBFs has outpaced research evaluating their composition, benefits, safety, and clinical outcomes (11). This single-center retrospective case series aimed to monitor gastrointestinal tolerance, glucose control, and weight change in children with hyperinsulinism who were prescribed FBF.

## 2 Materials and methods

### 2.1 Study design and patient population

This single-center retrospective case series reviewed the dietetic practice of prescribing a commercially available FBF (Compleat^®^ pediatric, Nestlé Health Science), in children (licensed for children one year old and over) with CHI and PPH. Compleat Pediatric is 1.2kcal/ml with 14% food‐derived ingredients containing rehydrated chicken, peas and green beans, with peach puree and orange juice; the fiber is from rehydrated vegetables 4.3% (pea fiber 3.8%, green beans 0.54%), acacia gum, fructo-oligosaccharide and inulin. Compleat Pediatric is not suitable for children with cow’s milk allergies or vegetarians.

### 2.2 Definition

Children who had been reviewed at our tertiary endocrine service with refractory hypoglycemia were included in this review. CHI diagnosis was based on documented hypoglycemia, in the presence of an intravenous glucose infusion rate of > 8 mg/kg/min, and/or detectable insulin or C-peptide levels with low free fatty acids and low ketone bodies (6). PPH was diagnosed with a positive mixed meal (liquid solution containing equal amounts of carbohydrate, protein and fat) and/or oral glucose tolerance test, which either elicited hypoglycemic symptoms or serum glucose levels reduced below 3.1mmol/L (55mg/dl) (6).

### 2.3 Data collection

Data was collected for all children prescribed an FBF via an enteral feeding tube at our specialist pediatric hospital between March 2022 and December 2024 (the timeframe FBF has been available at our specialist center). Parental consent was obtained from all participants, and the study was approved by the Great Ormond Street Hospital Audit, Quality Improvement and Service Evaluation Committee (registration number GOSH2024/4044). Children’s clinical and dietetic information was collected from the hospital’s electronic records (EPIC, Madison, WI, USA) and Electronic Dietetics Manager (MicroMan2000 Ltd) on demographics (age, sex ethnicity and primary diagnosis), feeding regimen (formula types and routes, carbohydrate intake, protein intake, fiber intake and protein-to-energy ratio), anthropometrics (weight and height-for-age Z-scores), hyperinsulinism related medication and glucose control. Data were collected one month before and one month after an FBF was prescribed. Data was extracted retrospectively, so we were only able to use documented medical and dietetic information – this consisted of carer-reported changes along with medical/dietetic observations.

### 2.4 Outcome measures

#### 2.4.1 Gastrointestinal symptoms

All children received nutritional assessments from a pediatric specialist endocrine dietitian. If gastrointestinal symptoms had been documented before commencing FBF we measured gastrointestinal symptoms as either improved, no change or worsened after FBF was prescribed on key markers of tolerance: (gastro-esophageal reflux, vomiting, constipation and loose stools). Constipation was defined by the Rome IV Criteria as less than three defecations a week and painful and hard stools. Loose stools were defined as more than one loose stool a day lasting longer than 7 days (12). Reflux was defined as the parental observation of the passage of gastric contents into the esophagus causing regurgitation, posseting, or vomiting, which leads to troublesome symptoms that affect daily functioning (13).

#### 2.4.2 Glucose monitoring

Dexcom Inc. Continuous Glucose Monitoring System^©^ (CGM) is a company that develops, manufactures, produces, and distributes continuous glucose monitoring systems for ambulatory glucose profiles. Continuous glucose monitoring allows close monitoring of glucose levels via a tiny electrochemical sensor electrode inserted under the skin (14). The sensor is connected to a transmitter, which sends this information to a detector, such as a smartphone, or a continuous subcutaneous insulin infusion (CSII) device and provides information about current and previous glucose levels, glucose trends and anticipated future glycemic status.

Dexcom generates a short report with statistical and graphic information, including glucose level statistics (e.g. hypoglycemia) and percentage glucose management indicator (GMI%). GMI% reports the patient’s approximate hemoglobin A1C level, based on the average glucose level from CGM readings for 14 or more days. In general, each 25 mg/dL (1.4mmol/L) increase in mean glucose corresponds to a GMI increase of 0.6%, e.g., a mean glucose of 150 mg/dL (8.3mmol/L) corresponds to a GMI of 6.9%. A healthy GMI target is below 7.5%. Dexcom also records the number of hypoglycemic events <63mg/dl (3.5mmol/L) and hyperglycemic events > 180mg/dl (10mmol/L/L) (15).

#### 2.4.3 Anthropometric measurements

Weight and height were extracted from EPIC electronic growth charts. Assessment of height reflects adequate growth and nutritional status but can be challenging in children with malformations. The nutrition status (weight-for-age and height-for-age) was assessed using Z-scores. Moderate overweight was identified if the Z-scores were between +2 and +3 standard deviations (SD) and severe overweight was identified if the Z-scores were above +3 (SD). Height and weight for age Z-scores were determined using the World Health Organization (WHO) growth standards, which describe the optimal growth of healthy children in a supportive environment (16).

### 2.5 Statistical analysis

Continuous data were tabulated using descriptive statistics (mean and standard deviation [SD]). To assess gastrointestinal tolerance of FBF, medical and dietetic reports of upper and lower gastrointestinal symptoms before starting FBF were compared with medical and dietetic reports of upper and lower GI symptoms within one month of FBF prescribed. Chi-square statistic was used to measure the probability of children being prescribed glucose polymer before and after FBF was prescribed; and to assess hypo and/or hyperglycemic episodes before and after FBF was prescribed. Comparative analysis was used to compare changes before and after (6 months and 1 month) the formula changed to FBF for weight-for-age Z-score, and nutritional composition. Statistical significance was based on an alpha level of 0.05. Data were analyzed using RStudio R version 4.3.2.

## 3 Results

### 3.1 Demographics

Data were collected on eight children, six were diagnosed with CHI and two were diagnosed with post-prandial hyperinsulinemia. Six children were female and two were male. The mean age when FBF was prescribed was 29 months (6.0SD). Six children were white British/European, and two children were Asian British (Pakistan). Five of the six children with CHI had the ABCC8 gene. All children had a gastrostomy (size 9 Freka - Fresenius Kabi) for enteral nutrition; case study 3 had a gastrostomy with a jejunal extension for the management of severe gastroesophageal reflux. Five of the eight children had some oral intake, which accounted for less than 20% of their estimated nutritional requirements (**Table 1**).

**Table 1:** Demographic, clinical and anthropometric data for children with hyperinsulinemia prescribed Food-based formula (FBF).

charactoriztics	1	2	3	4	5	6	7	8
Sex	Female	Female	Female	Female	Female	Female	Male	Male
Diagnosis	CHI	CHI	CHI	CHI	CHI	CHI	PPH	PPH
Ethnicity	White European	White British	Asian British	Asian British	White British	White British	White European	White British
Birth weight (kg)	3.5	4.7	3.85	4.1	4.37	4.2	3.8	4.2
Age at diagnosis (months)	12	2	1	1	2	2	12	16
Genetics	ABCC8	HNF4A	ABCC8	ABCC8	ABCC8	ABCC8	NA	NA
Current HI medication	Lanreotide	Diazoxide	Lanreotide/Diazoxide	Octreotide	Lanreotide	Lantreotide		Acarbose
Gastrointestinal symptom management medication	Lansoprazole	Omeprazole	Nil	Omeprazole	Nil	Senna	Lactulose Macrogel Omeprazole	Lansoprazole
Age when the formula changed to FBF (months)	36	24	24	12	34	49	13	36
Feeding route	Gastrostomy Oral	Gastrostomy Oral	Gastrostomy with jejunal extension	Gastrostomy	Gastrostomy and minimal oral	Gastrostomy and minimal oral intake	Gastrostomy	Gastrostomy Oral

### 3.2 Nutritional composition and feeding regimens

There was no significant difference before and after the formula changed to FBF for total fluids, kcal/day, carbohydrates (grams)/day, protein (grams)/day or Protein to Energy Ratio (**Table 2**). However, there was a significant difference in the amount of fiber intake before and after the formula changed to FBF, 95% CI -10, -0.9: p-value 0.03. (**Table 2**) Additionally, there was no significant difference before and after the formula changed to FBF for total fluids (**Table 2**); furthermore, feeding regimens were not altered after formula change – all children were on continuous feeds to optimize glucose control. Of note, there were no reported feeding tube blockages after the formula switch to FBF.

**Table 2:** Nutritional composition of enteral formula before and after Food based formula was prescribed.

Case study	Nutritional Information	1	2	3	4	5	6	7	8	Mean (SD)	P-value
Formula	Before	Paediasure Peptide and Vitajoule	21% Neocate Junior	21% Neocate Junior	16% Aptamil and Vitajoule	Peptijunior and Vitajoule	Paediasure and Vitajoule	Paediasure Fiber and vitajoule	Paediasure Plus Fiber		
Time on formula before switch (yrs)	Before	1.6	1.2	2	0.5	1.8	24	1.5	1.5	1.4 (0.5)	
Total fluids from feed (ml/day)	Before	780	800	700	1260	650	480	1000	200	790 (298)	
	After	690	700	820	1000	500	600	624	300	689 (196)	0.6
Kcal per day (kcal per 100ml)	Before	788 (101)	800 (100)	700 (100)	995 (79)	663 (100)	520 (1.1)	1005 (100)	300 (150)	764 (218)	
	After	807 (117)	819 (117)	959 (117)	1170 (117)	600 (120)	720 (1.2)	730 (117)	351 (117)	806 (229)	0.8
Carbohydrate grams per day (per 100ml)	Before	109 (14)	96 (12)	84 (12)	126 (10)	104 (16)	77 (16)	120 (12)	32 (16)	94 (29)	
	After	97 (14)	98 (14)	114 (14)	140 (14)	70 (14)	84 (14)	87 (14)	42 (14)	96 (27)	0.9
Protein, grams per day (per 100ml)	Before	23.4 (3)	22.4 (2.8)	19.6 (2.8)	17.6 (1.4)	11.7 (1.8)	13.4 (2.8)	28 (2.8)	8.4 (4.2)	20 (6.6)	
	After	25.0 (3.6)	25.2 (3.6)	29.5 (3.6)	36 (3.6)	18 (3.6)	21.6 (3.6)	22.4 (3.6)	10.8 (3.6)	25 (8.0)	0.1
Protein to Energy ratio, %	Before	11.5	11.2	11.2	7	7.1	9	11.2	14	11 (1.9)	
	After	12.3	12.3	12.3	12.3	12.3	12.3	12.3	12.3	12 (0)	0.2
Fiber grams per day (per 100ml)	Before	0	0	0	7.5 (0.6)	0	0	0.7	10 (1)	3.0 (3.8)	
	After	7 (1)	7 (1)	8.2 (1)	10 (1)	5 (1)	6 (1)	10 (1)	10 (1)	8.7 (1.3)	-0.03

Before the formula changed to a FBF, five children (cases 1, 4, 5, 6 and 7) were on additional glucose polymers to maintain blood glucose levels. After FBF commenced, all five children discontinued glucose polymers. The was a significant probability that children on FBF were less likely to require additional glucose polymer (Chi-square = 4.9, p-value = 0.02).

Five children’s feeding regimens were simplified after the formula changed to FBF; no longer requiring the need to add glucose polymer and/or transferred from a powdered amino acid/partially hydrolyzed formula to a ‘ready to feed’ formula (**Table 2**).

There were no changes to the oral intake in the four children who were able to tolerate solid food over the 6 months after FBF was commenced.

### 3.3 Gastrointestinal symptoms

All children had documented gastrointestinal symptoms before starting FBF, and of these six were prescribed proton pump inhibitors and/or stool softeners to mitigate gastroesophageal reflux and constipation symptoms (**Table 1**). Before FBF was prescribed, two children were on an amino acid formula and two children were on a partially hydrolyzed (peptide) formula due to upper gastrointestinal issues. The other three children were receiving a whole protein formula. Within one month of commencing FBF, the dietitians had reported an improvement in constipation symptoms in 4 of the eight children and an improvement in gastroesophageal reflux in 5 of the 8 children.

### 3.4 Anthropometrics

The mean weight-for-age Z-score when the FBF was prescribed = 0.18 (1.5 SD). Six months after the FBF was prescribed, the weight-for-age Z-score was 0.15 (1.6SD) (p-value 0.4). The mean weight (kg) increased from 12.3kg to 13.1kg over the 6 months children were prescribed FBF, p-value 0.08 (**Table 3**).

**Table 3:** Anthropometric changes from when FBF was prescribed and 6 months after commencing the Food-based formula (FBF).

Patient ID	1	2	3	4	5	6	7	8	Mean (SD)	P value
When FBF Prescribed										
Weight, Kg	14.4	10	10.7	14.8	13.0	15.2	11.4	12.4	12.3 (1.8)	
Weight- for- age Z-score	0.2	-1.2	-0.7	3.0	-0.2	-0.5	1.0	-1.2	0.18 (1.5)	
Height, cm	94.4	81.5	78.9	79	88.7	96	77	89.1	83.3 (6.3)	
Height-for-age Z-score	-0.3	-1.39	-2.29	1.9	-1.17	-0.9	-0.4	-1.89	-0.7 (1.4)	
6months after FBF Prescribed										
Weight, kg	15.5	12.9	11.8	14.7	13.9	15.6	11.6	12.1	13.1 (1.5)	0.08
Weight-for-age Z-score	0.4	-0.8	-0.7	3.0	-0.2	-0.5	1.0	-2.0	0.15 (1.6)	0.4

### 3.5 Glucose monitoring

**Figure 1:**
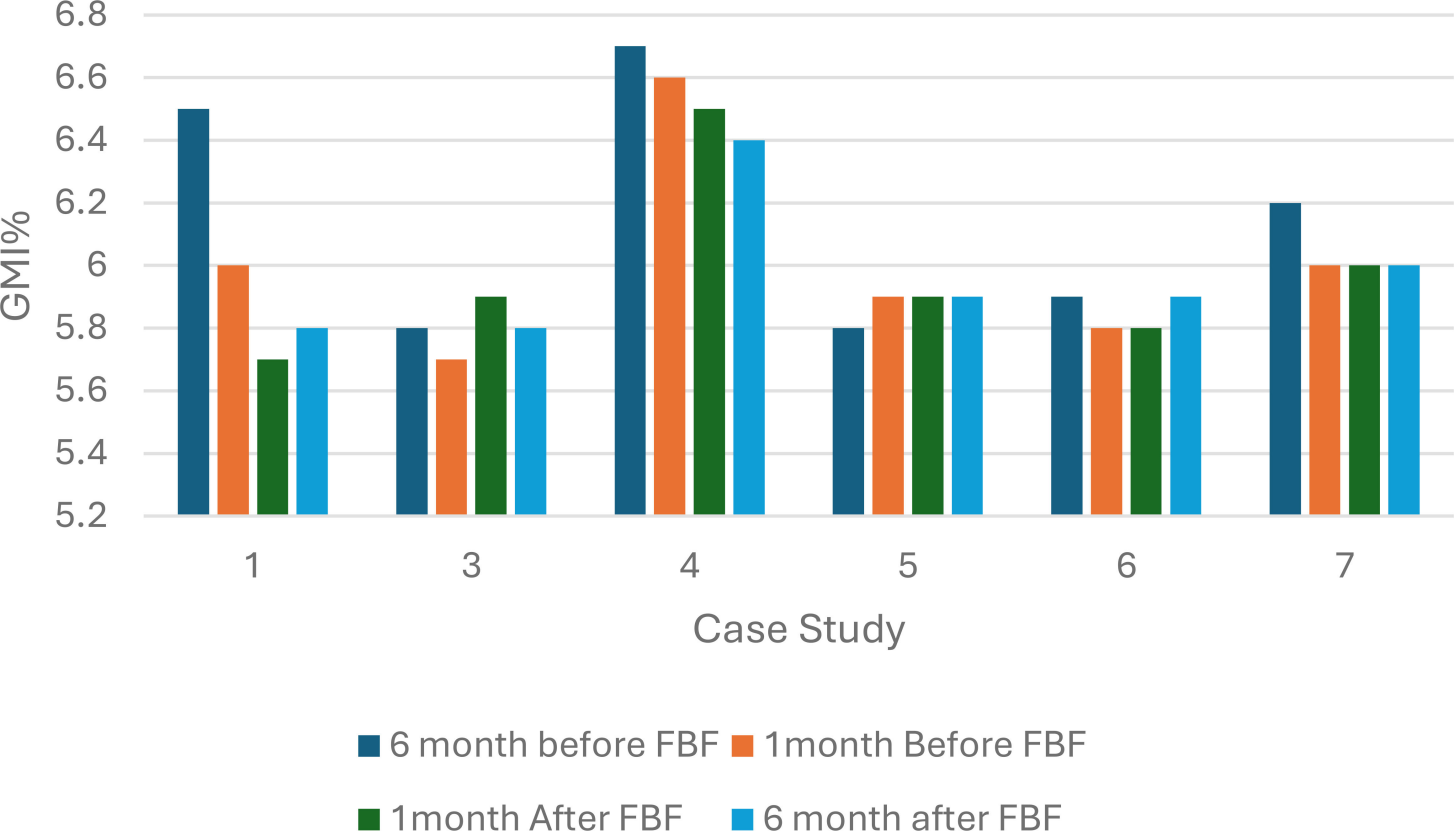
Percentage glucose monitoring indicator (%GMI) 6 months and 1 month before and after Food Based Enteral Formula prescribed in children with hyperinsulinism (n=6). FBF, Food Based Enteral Formula; %GMI, percentage glucose management indicator.

Continuous glucose monitoring data were available for six of the eight children; of these, five reported an improvement or stable GMI% one month after commencing FBF. Case studies 1 and 4 reported the greatest reduction in GMI%, 0.3% and 0.2%, respectively. Before the FBF was prescribed, both children were receiving additional glucose polymer. Case study 7 reported no change in GMI% before and after FBF was prescribed: this child was prescribed a high-energy whole protein formula with fiber before FBF. Case study 3 reported a 0.2% increase in GMI, before the formula change, this patient was receiving an amino acid formula (**Figure 1**). After 6 months on FBF case study 1,4 and 7 continued to report improved GMI%.

**Figure 2:**
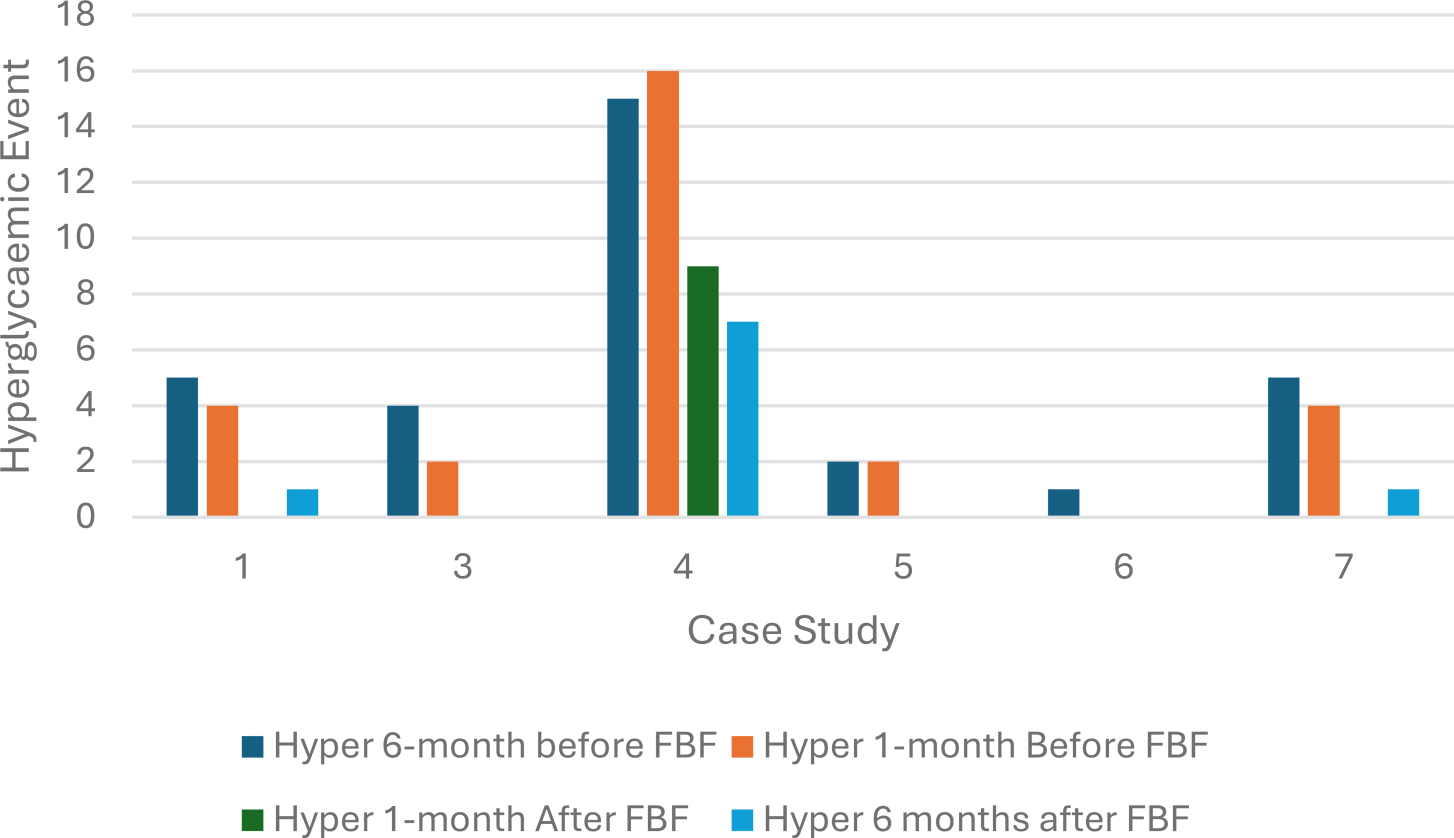
Hyperglycemic episodes at six months and one month before and after food-based formula was prescribed (n=6).

All six children had a decrease in the number of hyperglycemic episodes within one month after commencing FBF compared to one month before FBF was prescribed. This reduction in hyperglycemic episodes continued 6 months after the formula was switched to a FBF. The probability of having a hyperglycemic episode after FBF was prescribed was significantly reduced (chi-square statistic =5.8, p-value 0.02) (**Figure 2**).

**Figure 3:**
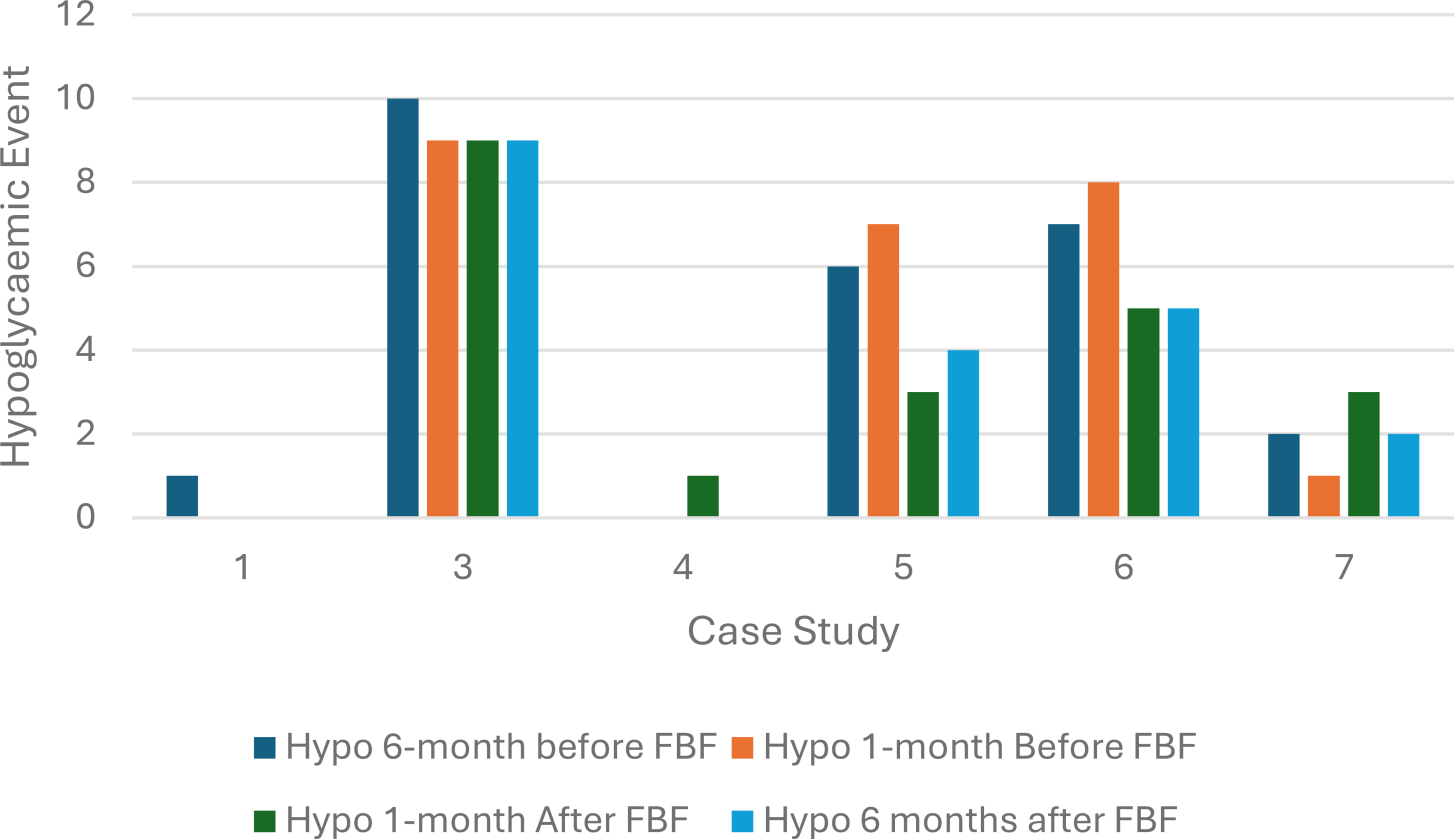
Hypoglycemic episodes at 6 and 1 month before and after food-based formula was prescribed (n=6).

Three children (case study 1, 3 and 5) had a reduction in hypoglycemic events, and two children had no change, while one child had an increase in hypoglycemia event. The probability of a hypoglycemic episode remained unchanged after FBF was prescribed (p-value =0.6) (**Figure 3**). 

## 4 Discussion

Our retrospective case series found that children with gastrointestinal symptoms (reflux and constipation) who were previously on an amino acid/hydrolyzed formula had improved symptoms after transitioning to a whole protein FBF. Also, children had a reduced reliance on glucose polymers when prescribed FBF. All children prescribed FBF had a significant reduction in hyperglycemic episodes. There was a significant increase in fiber intake after children were prescribed FBF.

Before commencing FBF, all eight children had documented gastrointestinal symptoms, with six children receiving medication to mitigate gastroesophageal reflux and constipation symptoms. Gastrointestinal symptoms were reported to have improved after the formula was changed to FBF. Previous retrospective studies that monitored the impact of FBF on gastrointestinal symptoms also reported a significant improvement in reflux and constipation (17, 18). In a community-focused review by Raskin et al. (2022), focused on increasing the understanding of the experience of living with CHI, families shared their own stories. Chronic constipation and stomach pain were commonly reported issues in children with CHI (19). Hyperinsulinism can cause gastric and intestinal motility deceleration, associated with reduced ghrelin and cholecystokinin levels, disturbing brain-gut axis functioning and may be responsible for gastric motility deceleration and subsequent constipation (20). Hyperinsulinism impairs gastrointestinal motility in both the postabsorptive and postprandial states - this effect is combined with delayed carbohydrate absorption. Therefore, hyperinsulinism may lead to alterations in carbohydrate absorption and can also contribute to gastrointestinal disturbances (21).

The average fiber intake in our case series significantly increased when the formula was changed to FBF, which may have contributed to the improved GMI% and reduction in hyperglycemic events. Of note, there was no difference in carbohydrate intake before and after the formula was changed to FBF. Dietary fiber is an essential component of the human diet and a major determinant of digestive health. Non-digestible dietary fiber undergoes fermentation by the intestinal microbiota to produce short-chain fatty acids (SCFAs), which positively impact the local and systemic immune system. A study by Xi et al. (2017) reported episodes of hypoglycemia significantly improved with the addition of fiber supplementation (22). Of note, a systematic review of randomized controlled trials investigating the clinical effects of fiber-supplemented enteral nutrition versus placebo in critically ill patients concluded that evidence suggests that fiber-supplemented enteral nutrition has clinical benefits, and recommends larger multi-center studies should be delivered to substantiate findings (23).

Carbohydrate metabolism of gut microbiomes has been proposed to contribute up to 10% of the host’s overall energy extraction (24). Fecal carbohydrates, particularly host-accessible monosaccharides, are increased in individuals with insulin resistance and are associated with microbial carbohydrate metabolisms and host inflammatory cytokines. A study by Takeuchi et al. (2023) investigated the relationship between gut microbiomes and glucose control using a comprehensive multi-omics strategy in humans. The authors concluded that gut bacteria were associated with insulin resistance and insulin sensitivity, which showed a distinct pattern of carbohydrate metabolism (25).

Although the exact mechanisms as to why FBF are better tolerated in some children remain unclear. One theory that has been postulated is associated with the amount and mixture of fiber and the subsequent beneficial impact on the gut microbiome (26). The diversity of the gut microbiome is influenced by the variety of the diet; a diet solely of commercial enteral feeds has been implicated in reducing the diversity of microbial species in the gut microbiome (27). Therefore, enteral formulas containing fiber may support normal digestive health. Commercial tube feeds devoid of fiber appear to negatively alter children’s gut microbiome (7).

After FBF was prescribed, all five children who were prescribed glucose polymers discontinued and three children transitioned from a partially hydrolyzed or amino acid powder formula to a whole protein ready-to-feed FBF. An estimated cost was calculated to assess the financial impact of transferring to an FBF. The average daily cost for 21% Amino acid formula with additional glucose polymer to 16% concentration at 1000ml/day = £40 per day (including additional ancillaries). Compared to 2 x 500ml ready-to-hang FBF bottles = £16/day. Transitioning to a FBF can equate to a cost saving to the health provider of £8760 per year. Furthermore, the reduced time for feed preparation after FBF was prescribed (estimated 15–20 minutes/day), reducing the disease burden of this complex disorder. Furthermore, the total feed volume decreased by an average of 100ml per day. This equates to a reduction of 1-1.5 hours on a feeding pump each day. A joint consensus guideline on gastroesophageal reflux reported evidence from a comparative study, which suggested that compared with larger volume feeds, smaller volumes are associated with fewer reflux episodes (13). This reduced time on the feeding pump may positively impact the child and family’s quality of life, with more time off the feeding pump and more time to play. Furthermore, reducing the feed volume may promote an increase in solid food, which can mitigate gastroesophageal reflux (28).

It is important to highlight that any child who has been tube-fed from birth will not have been through a weaning process and exposed to common allergens. Therefore, we would advise clinicians and parents to start by adding 30ml FBF with Food-derived ingredients to their current formula. Gradually building the volumes to tolerance and nutritional requirements. Detailed guidance is outlined in the British Dietetic Association’s Practice Toolkit: The Use of Blended Diet with Enteral Feeding Tubes, November 2021: Section 4.7.2 (29).

### 4.1 Limitations

Retrospective case series have several limitations owing to their design, which are dependent on the review of records and documentation and therefore the results are ungeneralizable rather than stating causation, we can only allude to a potential association that an FBF may improve gastrointestinal symptoms and glucose control in children with hyperinsulinism. The Glucose Management Indicator estimates Hemoglobin A1c (HbA1c) from continuous glucose monitoring data and is used in diabetes management, but it was not primarily created or validated for all people with diabetes or other blood glucose disorders. The standard GMI equation was developed from trials in adults with type 1 diabetes, and its performance, including its accuracy in predicting HbA1c, is not consistently well-characterized across different populations (30).

## 5 Conclusion

Our retrospective case series suggests that FBF is well tolerated in children with hyperinsulinism and may mitigate gut motility issues associated with hyperinsulinism, especially in relation to gastro-esophageal reflux and constipation symptoms. Furthermore, FBF may help reduce the dependency on glucose polymers and reduce the likelihood of a hyperglycemic episode. A larger sample size and longer follow-up study are necessary to substantiate the potential beneficial impact of FBF’s on glucose control in children with hyperinsulinism.

## Data Availability

The original contributions presented in the study are included in the article/supplementary material. Further inquiries can be directed to the corresponding author.
